# Examining the Impact of Food Security and Accessibility to Healthcare Services on Chronic Disease Risk Among Rohingya Refugees in Bangladesh

**DOI:** 10.3390/healthcare13040417

**Published:** 2025-02-14

**Authors:** Rizwanur Rahman, Fatema Afrouse, Md. Saiduzzaman Pulak, Md. Rabiul Karim, Mehjabin Haque, Mohammad Afshar Ali

**Affiliations:** 1 Department of Economics, Tongi Govt. College, National University Bangladesh, Gazipur 1711, Bangladesh; 2 Department of Economics, Faculty of Social Sciences, Jagannath University, Dhaka 1100, Bangladesh; miliafrouse2000@gmail.com (F.A.); rabiul@eco.jnu.ac.bd (M.R.K.); 3 Enterprise Development, Swisscontact, Dhaka 1212, Bangladesh; saiduzzamanpulak@gmail.com; 4 Department of Economics, Macquarie Business School, Macquarie University, Ryde, NSW 2113, Australia; 5 Department of Food and Nutrition, National College of Home Economics, Dhaka 1207, Bangladesh; mehjabinhaque.tanni@gmail.com; 6 School of Business, University of Southern Queensland, Toowoomba, QLD 4350, Australia; mohammadafshar.ali@usq.edu.au

**Keywords:** food security, healthcare access, chronic diseases, Rohingya refugees, Bangladesh

## Abstract

**Background:** Food security and access to healthcare are crucial determinants of health, but their impact on chronic disease risk among forcibly displaced populations is understudied. This study delves into the relationship between food security, accessibility to healthcare services, and chronic disease risk among Rohingya refugees in Bangladesh. **Methods:** Drawing from a nationally representative cross-sectional survey, this research investigates how the availability of food, accessibility to healthcare facilities, and utilization of services impact the likelihood of chronic diseases within this marginalized population. Using a cross-sectional survey collated from the UNHCR 2020 Joint Multi-Sector Needs Assessment survey, we deployed a series of multivariate logistic regression models to examine the relationship between food security, healthcare proximity, chronic disease, and sociodemographic characteristics. **Results:** Food security significantly decreased the risk of chronic diseases (OR = 0.65, 95% CI: 0.43, 0.98). Living far from healthcare facilities increased the risk (OR = 1.63, 95% CI: 1.03, 2.54). **Conclusion:** This study’s findings underscore the urgent need for targeted interventions aimed at enhancing food security and improving healthcare accessibility to alleviate the burden of chronic diseases among Rohingya refugees. By identifying key social determinants and barriers to healthcare access, this research equips policymakers with evidence-based strategies to design targeted interventions that improve nutrition, healthcare delivery, and chronic disease management for displaced populations.

## 1. Introduction

Conflicts, oppression, economic downturn, and environmental challenges have caused the displacement of 122 million people across the world where Rohingya refugees cover a significant portion [1]. In 2017, Bangladesh received 742,000 Rohingya refugees following a mass expulsion from Myanmar’s Rakhine State [2], who are living inside the world’s most densely refugee camp, resulting in one of the worst humanitarian crises for both migrants and host communities [3]. Arguably, a high concentration of forced migrants in certain areas results in a risk of food insecurity [4], leading to severe health conditions [5]. Health is a fundamental necessity for people worldwide, with food security serving as a vital component for ensuring overall well-being. Food security significantly influences both mental [6] and physical health, with impacts that extend from short-term outcomes to long-term consequences [7]. It is closely linked to the availability, accessibility, and regularity of food. Specifically, this includes ensuring adequate food availability in terms of quantity and quality, access to food for all individuals, the proper utilization of food, and stability in maintaining these factors over time. However, a considerable number of people across the world are vulnerable to food insecurity. Over 2 billion people experience moderate to severe food insecurity, which contributes to negative health outcomes, such as malnutrition, weakened immune systems, and chronic diseases [8,9].

Food security is a complex global challenge, particularly for forced migrants. Forced migration exacerbates food insecurity, which adversely affects not only the physical and mental health of migrants [6] but also all other aspects of their lives [7]. Additionally, a high concentration of forced migrants in certain areas places further strain on food security [4], leading to more severe health conditions [5]. In new living environments, forced migrants face elevated health risks due to the combined challenges of food insecurity [10] and limited access to healthcare services [11].

Lack of access to food degrades migrants’ current health conditions [5], while inadequate access to healthcare facilities significantly affects their future health outcomes [12]. Limited proximity to healthcare services often leaves migrants facing life-threatening health conditions [11], with many unable to access nearby facilities [13]. Moreover, barriers such as language differences, shortages of essential medications, and the physical distance to healthcare services—both within and outside migrant settlements—further hinder effective healthcare delivery [14]. These challenges compromise health services and heighten tensions between migrants and host communities [15]. In addition to these challenges, the lack of safe drinking water and other basic necessities complicates efforts to achieve better health outcomes [3]. This increases migrants’ vulnerability to food insecurity [4]. According to the World Health Organization (WHO), even preventable and treatable medical conditions can worsen without timely access to healthcare services [16]. Ultimately, poor living conditions, compounded by food insecurity and limited healthcare access, significantly heighten the risk of life-threatening diseases, including chronic illnesses.

Forcibly displaced people worldwide, including Rohingya refugees, face dire health conditions. Rohingya refugees have endured dreadful living conditions characterized by limited access to basic necessities and significant cultural barriers. Furthermore, the unfriendly attitudes of host communities and various forms of discrimination have exacerbated their plight [15]. The abysmal living conditions of Rohingya refugees have exposed them to a wide range of health risks, including both communicable and non-communicable diseases [3]. Among their unmet needs, food and healthcare remain two fundamental human rights, with their availability and accessibility being major concerns for Rohingya refugees in Bangladesh. Existing studies have highlighted negative health outcomes of food insecurity [4] and long-term health issues [9] especially on the mental and physical health of forcibly displaced people. For instance, research has demonstrated that food insecurity contributes to the prevalence of non-communicable diseases such as diabetes, hypertension, and cardiovascular diseases among refugee populations [3]. Those findings provide a clear foundation for understanding why addressing food security is critical for improving health outcomes. However, while there is broad consensus on the adverse mental health outcomes of food insecurity [6], the specific pathways through which food insecurity influences physical health outcomes, particularly chronic conditions, remain underexplored.

This study addresses significant gaps in the literature by focusing on the intersection of food security, healthcare access, and chronic disease risk in the context of forced migration. Specifically, it seeks to address the limited attention given to non-communicable diseases within refugee populations and the role that food insecurity and healthcare access play in influencing these health outcomes [17]. Additionally, this research contributes to the understanding of South–South migration and its implications for health service utilization—an area that has not been fully explored in previous studies [18]. Unlike the more extensively studied North–South migration patterns, South–South migration presents unique challenges and opportunities for health interventions, particularly in resource-constrained settings.

The primary objective of this study is to investigate the interconnectedness of food security, healthcare access, and the risk of chronic diseases among Rohingya refugees in Bangladesh. Using a nationally representative cross-sectional survey and rigorous statistical analyses, this study identifies key factors influencing the likelihood of developing chronic diseases within this population. By exploring the interaction between food security and healthcare access, this research will provide critical insights into how these factors jointly contribute to chronic disease risk [19]. Additionally, despite the widespread understanding of the adverse impacts of food insecurity on health, there is limited attention to how it interacts with access to healthcare services to influence the risk of chronic diseases in refugee populations. This research aims to provide a deeper understanding of these relationships and offer evidence-based recommendations to address these health challenges. By focusing on both physical and mental health outcomes, this study will contribute to the development of targeted interventions to reduce chronic disease risks and improve overall well-being in refugee settings.

The remainder of the paper is organized as follows: Section 2 describes the materials and methods employed in the study. Section 3 presents the results, while Section 4 focuses on discussing the findings. Finally, Section 5 offers policy recommendations and suggests potential avenues for future research.

## 2. Methods

### 2.1. Study Settings

The Rohingya, a Muslim ethnic minority, have lived in Myanmar for centuries, yet have not been recognized officially as an ethnic community of Myanmar. A vital indicator of their plight is their deprivation of voting rights, which makes them the largest stateless population in the world devoid of any basic rights [19]. Decades of oppression forced them to take refuge, especially in Bangladesh. In 2017, the largest exodus began; consequently, Bangladesh is now hosting nearly 1 million stateless Rohingya Refugees [2]. These Rohingya refugees are living in the world’s largest and most densely populated camps (Kutupalang and Nayapara) in Cox’s Bazar region, where 95% of them are dependent on humanitarian assistance [19]. This complete dependency of Rohingya refugees has only deteriorated their precarious conditions.

The key objective of this cross-sectional study was to provide a comprehensive evidence base of household-level multi-sectoral needs. By considering this issue, our study aims to explore how Rohingya refugees are exposed to the risk of chronic disease. Therefore, this research is a retrospective cross-sectional study using nationally representative survey data.

### 2.2. Questionnaire, Data Collection and Processing

The data were collected from households and individuals. While the household questionnaire consists of household information, education, health, food security, and livelihood-related questions, the individual questionnaire covers individuals’ information [19]. The socio-demographic variables, e.g., age, gender, employment, education, and household size, are categorized as follows: respondents’ answers regarding their age, gender, employment status, educational attainment, and household size. Employment was categorized based on the respondents’ responses to the questions related to income source, e.g., daily labor, own business, farming, assistance, begging, and none. Education was categorized based on the respondents’ responses to the questions related to formal schools, e.g., school, madrasah, technical college, university, and none (see Table 1 for more details and Appendix A for a snap shot).

Likewise, food security status was categorized into two categories: food security (Yes) and no food security (No). To assess this status, the questions focused on aspects such as purchasing food, receiving assistance, borrowing, and resorting to begging. Healthcare accessibility was categorized (near/far) based on the information decoded from the questions about access to health services. Conversely, the outcome variable ’Chronic disease risk’ was categorized as binary (yes/no). The chronic disease risk was measured through the answers to the question of ‘the respondent has critical/severe illness that needs medical treatment’.

From 27 July to 12 August 2020, data were collected by phone. Due to COVID-19 restrictions on movement, access to camps, and the need for preventative health measures, the survey was conducted using CATI (Computer-Assisted Telephone Interviewing). This method allowed for remote data collection, ensuring the safety of both participants and interviewers while still gathering accurate information. The use of telephone interviews was essential in overcoming the challenges posed by the pandemic, ensuring that the study could proceed while adhering to health guidelines. To conduct interviews, sector representatives conducted three days of online training for enumerators. Enumerators also underwent two-day piloting to familiarize themselves with the tools, protocols, and ethical issues. Before starting each interview, informed consent was sought, received, and documented. After collecting data each day, they were checked and cleaned according to the predetermined standard, e.g., outlier checks, correct categorization, and removal or replacement of inaccurate records [19].

### 2.3. Population and Sampling

The study population consists of 860,000 Rohingya refugees residing in 34 refugee camps across two sub-districts (Ukhiya and Teknaf) in Cox’s Bazar district, Bangladesh [19]. From the population figures available in UNHCR, target sample sizes for each camp were considered. Approximately 400 surveys were conducted per sub-district, and a total of 911 respondents were selected using stratified random sampling to ensure representation from various age groups, genders, and households. For example, during interviews over the phone, male- and female-headed households were sampled proportionately, although men are more prevalent in phone ownership.

### 2.4. Estimation Strategy

To examine the impact of food security and healthcare facility distance on chronic disease among Rohingya refugees, demographic characteristic data of the respondents were presented in terms of frequencies (N), and percentages (%). The results of the logistic regression analysis are reported as unadjusted and adjusted OR with 95% confidence intervals. For the adjusted model, the multivariate logistic regression estimates are enumerated after adjusting for standardized weights across the 911 cases. The statistical software ‘R’ (https://www.r-project.org/) was used for data cleaning, validation, and statistical computations.

The findings are presented through descriptive statistics, tables, and figures. Table 2 outlines the sociodemographic characteristics of the respondents. Table 3 illustrates the relationship between participant characteristics and chronic diseases. Regression estimates with unadjusted and adjusted odds ratios are presented in Table 4.

## 3. Results

The study results on chronic disease risk among Rohingya refugees are organized into three distinct but related themes. The themes are chosen for specific reasons: ‘background information and respondents’ will illustrate the socio-demographic characteristics of the Rohingya refugees, ‘relationship between chronic disease and participant characteristics’ will depict the significant relationship, and ‘factors affecting chronic disease’ will show how much the factors are statistically significant regarding having chronic disease.

### 3.1. Background Information and Respondents

The sociodemographic characteristics of the respondents are depicted in Table 2. A total of 911 participants were included in the study, with a nearly equal gender distribution, as 49.50% were female and 50.49% were male. The vast majority of respondents (98.02%) are under 50 years old, while only 1.98% are aged 50 or older. Regarding employment, 80.02% of respondents are employed and 19.98% are not. In terms of education, 67.50% have received formal education while 32.49% have not.

In terms of household size, 62.89% of respondents reported living in households with eight or more members, while 37.10% had fewer than eight members. A notable finding was that 79.36% of respondents lived far from healthcare facilities, indicating challenges in accessing healthcare services. Furthermore, 87.48% of the respondents reported having no chronic disease risk, while 12.51% were at risk of chronic illness. A concerning result was that 34.02% of the participants experienced food insecurity.

### 3.2. Relationship Between Chronic Disease and Participant Characteristics

Table 3 shows the relationship between chronic disease risk and various socio-demographic characteristics of the participants. The findings reveal that age, gender, employment status, education, household size, and food security do not have statistically significant associations with chronic disease risk. For example, the *p*-values for forage (0.0920), gender (0.3989), education (0.2457), household size (0.7192), employment (1.0000), health distance (0.0484), and food security (0.1033) are all greater than the threshold of 0.05, suggesting no significant relationship between these factors and chronic disease risk in this study. On the other hand, the distance to healthcare facilities shows a significant association with chronic disease risk, as indicated by the *p*-value of 0.0484. This suggests that participants who live farther from healthcare centers are more likely to be at risk for chronic diseases. Household size also did not show a significant relationship, with a *p*-value of 0.7192, indicating no effect on chronic disease risk. These results highlight the importance of access to healthcare facilities as a potential factor influencing chronic disease risk, while other socio-demographic characteristics appear to have little impact.

### 3.3. Factors That Affect Chronic Diseases

The results of unadjusted and adjusted odds ratios (ORs) for regression estimates of chronic diseases with a 95% CI are depicted in columns (1) and (2), respectively, of Table 4. The results indicate that distance to healthcare facilities and food security are the most significant factors influencing chronic disease risk. In both the unadjusted and adjusted models, individuals living far from healthcare facilities have a significantly higher likelihood of having chronic disease risk, with an OR of 1.60 (95% CI: 1.01–2.47) in the unadjusted model and 1.63 (95% CI: 1.03–2.54) in the adjusted model. This suggests that limited access to healthcare is a notable factor contributing to chronic disease risk. Additionally, those with food security have a significantly lower risk of chronic diseases. The unadjusted OR is 0.70 (95% CI: 0.47–1.05) and the adjusted OR is 0.65 (95% CI: 0.43–0.98), indicating that food-secure individuals are less likely to face chronic disease risks.

In contrast, other factors such as age (OR: 1.66, 95% CI: 0.86, 1.10), gender (OR: 1.38, 95% CI: 0.93, 2.08), employment (OR: 1.02, 95% CI: 0.62, 1.73), education (OR: 0.74, 95% CI: 0.46, 1.16), and household size (OR: 0.85, 95% CI: 0.56, 1.28) do not show statistically significant associations with chronic disease risk. This suggests that, after adjusting for other variables, these factors do not significantly contribute to the risk of chronic diseases in this population. Overall, the analysis highlights the importance of access to healthcare and food security in influencing chronic disease risk, while other socio-demographic factors seem to have no substantial impact.

We found that age and gender do not significantly impact the odds of chronic diseases. Compared to the baseline age group below 50 years, the age group of 50 years and above or 50 (OR: 1.66, 95% CI: 0.86, 1.10) possess higher chances of having a chronic disease. Although the result has shown a potential age-related vulnerability, the overall impact of age on the outcome variable was not significant. Men (OR: 1.38, 95% CI: 0.93, 2.08) have a higher adjusted odds ratio for chronic diseases than women. The magnitude of this difference is relatively small and does not have a significant effect on the odds of chronic disease. Surprisingly, the model has found that household income (OR: 1.02, 95% CI: 0.62, 1.73) and household size (OR: 0.85, 95% CI: 0.56, 1.28) do not have a substantial impact on chronic diseases. Thus, this implies that employment and household size might not be significant influencing factors that determine the odds of chronic diseases.

Education (OR: 0.74, 95% CI: 0.46, 1.16) has no critical impact on the risk of having chronic diseases. However, the distance to a healthcare center increases the odds of getting treatment by 63% (OR: 1.63, 95% CI: 1.03, 2.54) compared to respondents living close to the healthcare center, which is significantly associated with the risk of chronic diseases. Likewise, for households with no food security, there is a 35% higher chance of chronic diseases (OR: 0.65, 95% CI: 0.43, 0.98) compared to households with an adequate supply of food, which is a significant association.

To summarize, factors including age (OR: 1.66, 95% CI: 0.86, 1.10), gender (OR: 1.38, 95% CI: 0.93, 2.08), employment (OR: 1.02, 95% CI: 0.62, 1.73), household size (OR: 0.85, 95% CI: 0.56, 1.28), and education (OR: 0.74, 95% CI: 0.46, 1.16) have no significant association with chronic disease while. The results revealed that people living far from healthcare facilities had a greater risk of chronic disease (OR = 1.63, 95% CI: 1.03, 2.54. Conversely, food security reduces the risk of chronic disease.

## 4. Discussion

This study provides significant insights into the interplay between food security, healthcare access, and chronic disease risk among Rohingya refugees, offering a valuable contribution to the limited research on the health outcomes of refugee populations in South–South migration contexts. By focusing on the unique challenges faced by Rohingya refugees in Bangladesh, this research highlights structural factors—rather than conventional sociodemographic determinants—as critical drivers of chronic disease risk. This novel perspective adds depth to the broader understanding of health disparities among forcibly displaced populations and their implications for public health interventions. More importantly, our study sheds light on a different narrative within the landscape of health service utilization by highlighting the role of South–South migration. Specifically, we examined healthcare utilization behaviors in the context of humanitarian migration between developing countries—namely, from Myanmar to Bangladesh—rather than the more commonly studied migration from developing to developed nations.

Our findings demonstrate that food insecurity significantly increases the likelihood of chronic diseases among Rohingya refugees, underscoring the importance of adequate and diverse food supplies. Consistent with prior studies [8,9,21], we confirm that insufficient nutrition exacerbates health vulnerabilities, particularly in marginalized communities, by increasing the risk of non-communicable diseases [23]. For instance, malnutrition not only affects immediate well-being but also has long-term repercussions, including heightened susceptibility to chronic illnesses. This aligns with existing evidence [25] that improving dietary diversity and ensuring food security are essential strategies for mitigating health risks.

Similarly, our results reveal that refugees residing farther from healthcare facilities face higher risks of chronic diseases, emphasizing the critical role of accessibility in health outcomes. Access to healthcare enables timely interventions, routine check-ups, and the management of early symptoms, which are crucial for preventing chronic conditions. Previous studies [11,13,26] have shown that distance to healthcare facilities is a significant barrier to care, particularly for vulnerable populations. This study extends this knowledge by highlighting its implications within the context of forced migration and resource-scarce environments, where healthcare availability is often limited.

Unexpectedly, traditional sociodemographic factors such as age, gender, and employment status did not significantly impact chronic disease risk in this population. This finding diverges from established research [21,23], which typically associates older age, male gender, and unemployment with higher rates of chronic conditions. One explanation behind this counterintuitive finding may lie in the relatively small and homogeneous nature of the survey population. Given that most participants were under 50 years old and resided in similarly precarious conditions, the variability within the sample may have been insufficient to detect significant differences. Furthermore, structural determinants such as inadequate food and healthcare access may overshadow individual-level demographic influences in refugee contexts.

This study has several limitations that should be acknowledged. First, the cross-sectional design precludes causal inferences about the relationships between food security, healthcare access, and chronic disease risk. Longitudinal studies would provide a clearer understanding of how these factors evolve. Second, the relatively young age distribution of the sample limits the generalizability of findings to older populations, where chronic disease prevalence is typically higher. Third, the self-reported nature of health data may introduce reporting biases, as participants might under-report or over-report health conditions due to stigma or misunderstanding. Additionally, the study focuses exclusively on the Rohingya population, and findings may not be generalized to other refugee populations with differing contexts and experiences.

### Future Research Direction

This study underscores significant gaps in addressing chronic disease risks among refugee populations, particularly in resource-constrained settings like the Rohingya refugee camps in Bangladesh. To build on these findings, both stakeholders and researchers must prioritize future work that addresses immediate and long-term strategies to mitigate these health risks.

For stakeholders, enhancing food security remains a critical priority. This includes developing sustainable food supply systems, integrating culturally appropriate diets, and introducing nutritional education programs to reduce the risks of chronic diseases linked to food insecurity. Similarly, improving healthcare accessibility is essential. Establishing mobile health clinics, expanding community-based health programs, and building more healthcare facilities closer to refugee settlements can facilitate timely care, enabling regular check-ups, early disease detection, and effective chronic condition management. Governments and humanitarian organizations should also collaborate to design inclusive health policies tailored to refugees’ unique challenges. Such policies should integrate refugee health into national health systems, secure funding for specialized interventions, and address barriers like language and cultural differences.

For researchers, conducting longitudinal studies can provide deeper insights into how food insecurity and healthcare access evolve over time and their cumulative effects on chronic disease risk. Exploring psychosocial and environmental factors, such as stress, trauma, and living conditions, is another critical avenue for understanding the broader determinants of health outcomes in refugee populations. Additionally, assessing the effectiveness of targeted interventions, such as nutritional supplementation programs and mobile healthcare initiatives, can help determine which strategies yield the best results. Comparative studies across different refugee contexts could further highlight common challenges and context-specific needs, offering valuable lessons for global health policy and practice. Expanding the focus beyond chronic diseases to include other pressing health issues, such as mental health disorders and infectious diseases, will also contribute to the development of comprehensive health strategies.

By addressing these areas, future research and policy efforts can build on this study’s findings, contributing to improved health outcomes and a better quality of life for refugee populations worldwide.

## 5. Conclusions

The Rohingya refugee crisis is one of the worst man-made crises in the world and has displaced many people and created one of the worst humanitarian crises in history. Rohingya refugees are living under abysmal living conditions, which have exposed them to various health risks. Health risks become much worse when they face food insecurity. Studies have found that the risk of chronic disease is much higher in food-insecure conditions, yet this could have led to worse conditions without the proximal distance of healthcare facilities. Notably, it has been observed that age, gender, income, and household size have no impact or a minor impact on chronic disease, which requires further investigation. This study suggests necessary policy interventions to address the challenges of chronic health risks among Rohingya migrants in Bangladesh. Moreover, future studies using longitudinal follow-up data are suggested, which may help to understand the natural progression of Rohingya refugees’ chronic health risks.

## Figures and Tables

**Table 1:** The variables included in our statistical model.

Variable Name	Definition & Significance	Reference
*Independent variables*		
Age	Age is an important factor in developing chronic diseases especially those in the higher age group. The model includes this variable to determine whether age detrimentally affects the migrants’ chronic health. The model coded the respondents’ answers as (Less than 50 = ‘0’ & More than or equal to 50 = ‘1’).	[20]
Gender	Social factors in socially disadvantaged areas play a significant role in cardiovascular diseases across genders. This variable is a crucial confounding factor that may distort the results of our study. The model coded the respondents’ answers as (Female = ‘0’ & Male = ‘1’)	[21]
Employment	Due to lower household income, people often face difficulties in accessing healthcare which leads to poorer health outcomes, especially in chronic conditions. It is incorporated into the model to understand the impact of income on Rohingya refugees’ chronic health. This variable was categorized in the model as (Not employed = ‘0’ & Employed = ‘1’).	[22,23]
Household size	Larger household sizes have potentially lowered the resource distribution among family members which may cause negative health outcomes. The model includes this variable to understand how household size affects Rohingya refugees’ chronic health. This variable was coded in the model as (Less than 8 = ‘0’ & More than or equal to 8 = ‘1’).	[24]
Education	People across socially disadvantaged areas have used to live under abysmal living conditions. Due to a lack of education migrants have potentially faced difficulties in communicating and getting enough information. It is integrated into the model to identify the potential reason behind increasing the risk of chronic health of Rohingya refugees. The model coded the respondents’ answers as (Formal learning = ‘0’ & No formal learning = ‘1’).	[3,14]
Distance to healthcare facility	Timely access to healthcare services is essential to maintaining a healthy life and lowering the risk of facing critical health conditions. This variable is included to examine the health outcomes of the Rohingya refugees. The model categorized this variable as (Near = ‘1’ & Far = ‘2’) where refugees living less than 15 km had been coded as ‘Near’ and more than 15 as ‘Far’.	[11,16]
Food security	Food insecurity weakens the immune system and leads to negative health conditions, especially risking chronic health. The model integrates this variable to determine the chronic health vulnerability among Rohingya refugees. The model coded this variable as (No food security = ‘0’ & Has food security = ‘1’).	[8,9]
*Outcome Variables*		
Chronic disease risk	Food insecurity and limited healthcare access heightens the risk of having chronic diseases among Rohingya refugees. This variable captures the critical reason for identifying the risk of having chronic diseases among Rohingya refugees. It was categorized as binary (No chronic disease risk = ‘0’ & Has chronic disease risk = ‘1’)	[3,10,15]

**Table 2:** Distribution of respondents’ socio-demographic characteristics.

Characteristics	Observation (N = 911)	Percentage (%)	95% CI	
			Lower Limit	Upper Limit
**Age**				
<50	893	98.02	96.88	98.75
≥50	18	1.98	1.24	3.11
**Gender**				
Female	451	49.50	46.25	52.75
Male	460	50.49	47.24	53.74
**Employment**				
Not Employed	182	19.98	17.50	22.70
Employed	729	80.02	77.29	82.49
**Education**				
Formal learning	615	67.50	64.39	70.47
No Formal Learning	296	32.49	29.52	35.60
**Household size**				
<8	338	37.10	34.01	40.29
≥8	573	62.89	59.70	65.98
**Distance to healthcare facility**				
Near	188	20.63	18.12	23.39
Far	723	79.36	76.60	81.87
**Food security**				
No	310	34.02	31.01	37.17
Yes	601	65.97	62.82	68.98
**Chronic disease risk**				
No	797	87.48	85.17	89.48
Yes	114	12.51	14.82	10.51

**Table 3:** Relationship between risk of chronic diseases and participant characteristics.

Characteristics	Observation (N = 911)	Percentage (%)	95% CI		*p*-Value
			Lower Limit	Upper Limit	
**Age**					
<50	893	98.02	96.88	98.75	0.0920
≥50	18	1.98	1.24	3.11	
**Gender**					
Female	451	49.51	46.25	52.75	0.3989
Male	460	50.49	47.24	53.74	
**Employment**					
Not Employed	182	19.98	17.50	22.71	1.0000
Employed	729	80.02	77.29	82.49	
**Education**					
Formal learning	615	67.51	64.39	70.47	0.2457
No Formal Learning	296	32.49	29.52	35.61	
**Household Size**					
<8	338	37.10	34.01	40.29	0.7192
≥8	573	62.89	59.71	65.98	
**Health Distance**					
Near	188	20.63	18.12	23.39	0.0484
Far	723	79.36	76.60	81.87	
**Food Security**					
No	797	87.48	85.17	89.48	0.1033
Yes	114	12.51	14.82	10.51	

**Table 4:** Factors that affect risk of chronic diseases.

	Unadjusted Model (1)				Adjusted Model (2)			
Characteristics	OR ^1^	RSE ^2^	95% CI		OR ^1^	RSE ^2^	95% CI	
			Lower Limit	Upper Limit			Lower Limit	Upper Limit
**Age**								
<50	1.00	-	-	-	1.00	-	-	-
≥50	1.79	0.30	0.95	3.20	1.66	0.32	0.86	1.10
**Gender**								
Female	1.00	-	-	-	1.00	-	-	-
Male	1.35	0.20	0.91	2.01	1.38	0.20	0.93	2.08
**Employment**								
Not Employed	1.00	-	-	-	1.00	-	-	-
Employed	1.05	0.25	0.65	1.76	1.02	0.25	0.62	1.73
**Education**								
No Formal learning	1.00	-	-	-	1.00	-	-	-
Formal Learning	0.71	0.22	0.45	1.09	0.74	0.23	0.46	1.16
**Household Size**								
<8	1.00	-	-	-	1.00	-	-	-
≥8	1.05	0.25	0.65	1.76	0.85	0.20	0.56	1.28
**Distance to healthcare facility**								
Near	1.00	-	-	-	1.00	-	-	-
Far	1.60 *	0.22	1.01	2.47	1.63 *	0.23	1.03	2.54
**Food security**								
No	1.00	-	-	-	1.00	-	-	-
Yes	0.70 *	0.20	0.47	1.05	0.65 *	0.20	0.43	0.98

^1^ OR: Odd Ratio; ^2^ RSE: Residual Standard Error; ‘*’ Significant result in terms of 5% Confidence Interval.

**Figure A1:**
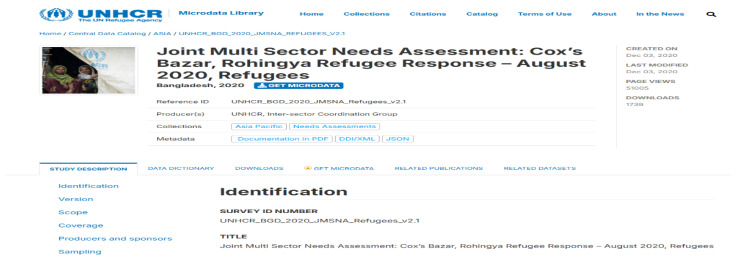

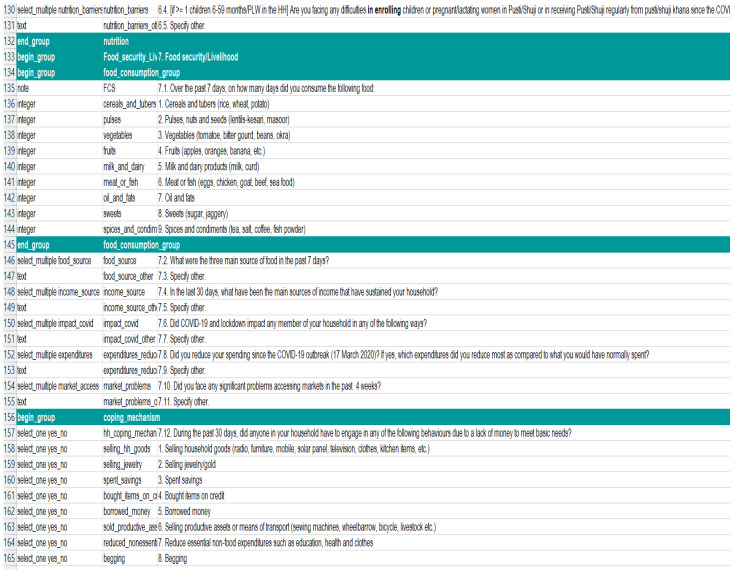
Source: [19] For more information please explore UNHCR microdata library: https://microdata.unhcr.org (accessed on 30 April 2023).

## Data Availability

This paper uses unit record data from ‘Joint Multi Sector Needs Assessment: Cox’s Bazar, Rohingya Refugee Response 2021, Host Community’ survey conducted UNHCR. Access to this restricted dataset requires a formal application. For more information on obtaining access to the data, please visit the following website: https://microdata.worldbank.org/index.php/catalog/unhcr/?page=1&ps=15&repo=unhcr (accessed on 30 April 2023). The list of selected procedures and the extracted data are available on request.
